# Measurement-free, scalable, and fault-tolerant universal quantum computing

**DOI:** 10.1126/sciadv.adv2590

**Published:** 2025-08-13

**Authors:** Friederike Butt, David F. Locher, Katharina Brechtelsbauer, Hans Peter Büchler, Markus Müller

**Affiliations:** ^1^ Institute for Quantum Information, RWTH Aachen University, Aachen, Germany.; ^2^ Institute for Theoretical Nanoelectronics (PGI-2), Forschungszentrum Jülich, Jülich, Germany.; ^3^ Institute for Theoretical Physics III and Center for Integrated Quantum Science and Technology, University of Stuttgart, Stuttgart, Germany.

## Abstract

Reliable execution of large-scale quantum algorithms requires robust underlying operations, which is addressed by quantum error correction (QEC). Most modern QEC protocols rely on measurements and feed-forward operations, which are experimentally demanding and often prone to high error rates. Additionally, no single-error–correcting code intrinsically supports the full set of logical operations required for universal quantum computing. In this work, we present a complete toolbox for fault-tolerant universal quantum computing without measurements during algorithm execution by combining the strategies of code switching and concatenation. We develop fault-tolerant, measurement-free protocols to transfer encoded information between 2D and 3D color codes that offer complementary and, in combination, universal sets of robust logical gates. Moreover, we extend the scheme to higher-distance codes by concatenating the 2D color code and integrating code switching for operations lacking a natively fault-tolerant implementation. Our measurement-free approach thereby provides a practical and scalable pathway for universal quantum computing on state-of-the-art quantum processors.

## Teaser

A method for scalable, fault-tolerant quantum computing without measurements is outlined.

## INTRODUCTION

A key requirement for the practical deployment of quantum algorithms is their robustness against noise, alongside the capacity to implement arbitrary operations on qubits. Quantum error correction (QEC) provides protection against noise by enabling the detection and correction of errors that arise during computation (*1*), and recent experiments have demonstrated substantial breakthroughs in the field (*2*–*5*). The latter is realized through a discrete, universal set of gates capable of approximating any quantum operation to in principle arbitrary precision (*1*). Fault-tolerant (FT) implementations of these gates prevent the uncontrolled propagation of errors through suitable quantum circuit design principles (*6*). However, achieving such FT implementations of a full universal gate set poses a substantial challenge, as no known QEC code intrinsically supports a fully FT universal gate set (*7*). Two well-established methods to complete an FT universal gate set are magic state injection and code switching. Magic state injection makes use of a fault-tolerantly prepared logical magic resource state (*8*), which is injected onto the encoded data qubit (*9*). Code switching enables the combination of two codes with complementary sets of transversal gates by transferring encoded information between them (*10*, *11*). Recent experiments have demonstrated an FT universal gate set by means of code switching (*12*) and FT computations in combination with error correction (*3*, *5*, *13*, *14*). However, the success probability of many practical protocols is fundamentally limited by mid-circuit measurements, which is challenging on many hardware platforms. For instance, in atomic setups, such as trapped ions and neutral atoms, fluorescence measurements heat up the atoms, which require additional laser cooling during or after the read-out. Moreover, in atomic as well as superconducting quantum processors, measurements are still orders of magnitude slower than typical gate times, which lead to decoherence of idling qubits in the meanwhile and imposes severe speed limitations (*4*, *12*, *15*–*17*). Real-time decoding (*3*, *4*) and feedback based on measurement outcomes has been realized but is still experimentally demanding (*3*, *18*). In contrast to this, resetting qubits can typically be done fast nondestructively. These limitations and experimental capabilities motivate the search for FT protocols that do not rely on mid-circuit measurements or feed-forward operations. Recently, measurement-free (MF) schemes for state preparation (*19*) and QEC on different codes have been constructed (*20*–*22*). The idea behind MF QEC schemes, as summarized in (*23*), is to transfer the stabilizer information onto additional auxiliary qubits and perform decoding as well as coherent feedback within the quantum algorithm itself. Last, auxiliary qubits can be reset to be reused or substituted with fresh qubits, which effectively removes the entropy introduced by the noise.

While the principal idea that MF logical operations are possible has been outlined in earlier works (*23*, *24*), so far a concrete scheme to implement an FT universal gate set without relying on measurements, the existing MF schemes only consider small, low-distance code instances, such as the nine-qubit Bacon-Shor code (*22*, *24*) or the seven-qubit color code (*20*, *21*, *23*), and do not provide a general method to scale this approach to larger-distance codes with increased protection and compatibility with computational universality.

**Fig. 1.**
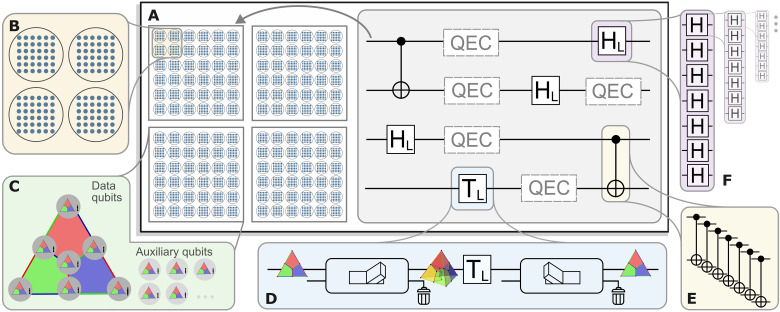
MF universal quantum computing by means of concatenation and code switching. (**A**) A quantum algorithm can be constructed from a universal logical gate set such as {$H_{L}$, ${\text{CNOT}}_{L}$, $T_{L}$} and QEC to maintain fault tolerance in an algorithm. We provide a complete toolbox to run these circuits fault-tolerantly on logical qubits, which are encoded in blocks of physical qubits. (**B**) Physical qubits form logical qubits, which, in turn, again encode logical qubits, a scheme known as concatenation. (**C**) We choose the seven-qubit color code as the base-code of our protocols, which is concatenated with itself and requires a set of auxiliary qubits. (**D**) To apply the logical gate $T_{L}$, we switch to a 3D color code that has a transversal implementation. Afterward, the encoded quantum information is transferred back to the initial code. (**E** and **F**) The Clifford operations $H_{L}$ and ${\text{CNOT}}_{L}$ can be performed transversally on the [[7,1,3]] code and, thus, in a natively FT way by bitwise application of the respective physical operations.

In this work, we show how quantum computers can be run autonomously, without measurement interventions, freely programmable, and yet in an FT manner. We achieve this by developing a scheme for freely scalable FT and MF quantum computing that combines code switching and code concatenation. First, we construct MF FT code switching schemes to transfer encoded information between the smallest instances of a two- (2D) and a three-dimensional (3D) color code. This enables the implementation of a deterministic FT universal gate set that does not require measurements or feed-forward operations during the execution of a logical quantum algorithm. Then, we scale our schemes to high distances by concatenating a code block with itself and including switches, as illustrated in Fig. 1.

## RESULTS

**Fig. 2.**
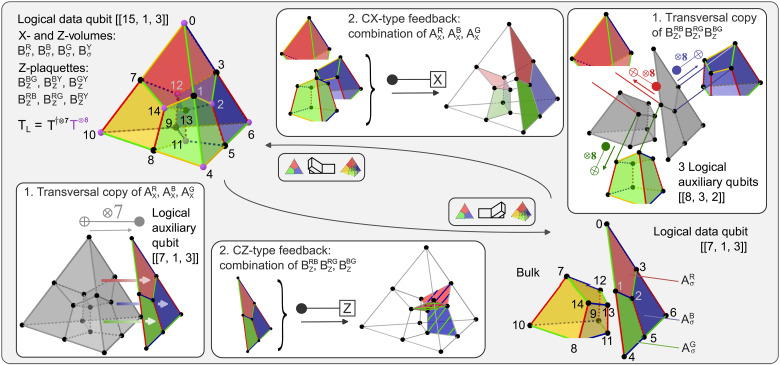
MF fault-tolerant code switching. We can switch between the [[15, 1, 3]] and the [[7, 1, 3]] code fault-tolerantly and without any measurements by making use of auxiliary logical qubits and controlled Pauli operations. A logical auxiliary qubit is initialized in the $|\;0\rangle_{L}$ state of the [[7, 1, 3]] code for switching to the [[7, 1, 3]] code. Then, the X-stabilizers of the target code are mapped out by means of a transversal CNOT gate and stored in a subset of physical auxiliary qubits. Then, a combination of controlled Z operations is applied to fix the state into the correct codespace. A similar strategy is used for the inverse switching direction: The [[8, 3, 2]] code is a convenient choice to map out the desired stabilizer, and we initialize three logical auxiliary qubits in the $|\;+++\;\rangle_{L}$ state of the [[8, 3, 2]] code. The target Z-stabilizers are then copied to the auxiliary logical qubit with transversal CNOT gates. Last, controlled Pauli-X operations are applied to implement the switching operation.

**Fig. 3.**
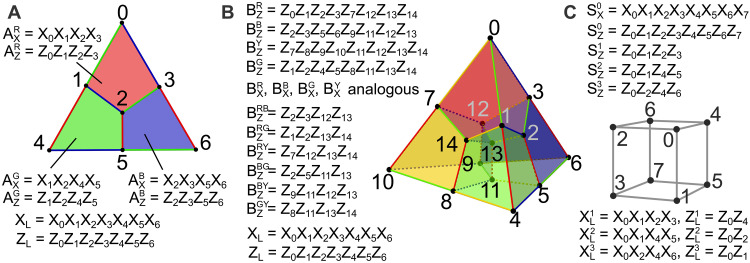
Stabilizer operators of codes used for MF FT code switching. (**A**) The X- and Z-stabilizers of the [[7, 1, 3]] color code are defined symmetrically on the red, blue, and green plaquettes (*25*). (**B**) Four X-stabilizers of the [[15, 1, 3]] code have support on the eight qubits which form one cell. Four Z-stabilizers are defined analogously on these four cells as well as six additional independent Z-stabilizers on the weight 4 interfaces between cells (*26*, *58*). (**C**) One X- and Z-stabilizer of the [[8, 3, 2]] code each has support on all eight qubits. Three additional Z-stabilizers are defined on the faces of the cube (*59*).

The smallest instance of the 2D color code [[n=7,k=1,d=3]], commonly known as the Steane code, encodes a single logical qubit k=1 in seven physical qubits n=7 and has distance d=3, meaning that any single error can be corrected (*25*). Three X- and Z-stabilizers are defined symmetrically on the plaquettes formed by four physical qubits, as illustrated in Figs. 2 and 3B. The logical Pauli operators correspond to applying X- and Z-operations to all seven qubits and a logical Hadamard operation can be implemented transversally by applying seven single-qubit Hadamard gates. In 3D, the smallest error-correcting instance is the tetrahedral [[n=15,k=1,d=3]] color code, also known as a Reed-Muller code. It encodes k=1 logical in n=15 physical qubits and has distance d=3 (*26*). The stabilizer generators of this code are given in Figs. 2 and 3B, and the logical X- and Z-operators of this code coincide with those of the seven-qubit color code. The X- and Z-stabilizers are defined on different support, thus not allowing the transversal Hadamard gate. However, a transversal FT non-Clifford T gate can be implemented by applying physical T- and T^†^-operations in a predefined pattern to all 15 qubits. The combination of these fully transversal gates, together with FT code switching, gives rise to a fully transversal universal gate set. We first review the existing code switching procedure, before discussing the extension to an MF setting.

### Measurement-based code switching

With measurement-based code switching (*11*, *12*, *27*, *28*), one can transfer encoded information between the two codes introduced above by, first, measuring the subset of stabilizers of the target code, which are not shared with the initial code. This randomly initializes the state in a +1- or −1-eigenstate of the measured operators. Second, local Pauli generators are applied to bring the state into a +1-eigenstate of all target stabilizers without changing the logically encoded information (*10*, *11*). Specifically for switching from [[15, 1, 3]] to [[7, 1, 3]], we measure the three X-stabilizers $\left(A_{X}^{R},A_{X}^{B},A_{X}^{G}\right)$ of [[7, 1, 3]] as shown in Fig. 2. The initial tetrahedral code is a +1-eigenstate of the weight 8 cells, so this measurement yields random outcomes ±1 for each target stabilizer. Next, a combination of the Z-stabilizers connecting the [[7, 1, 3]] instance with the yellow cell is applied, i.e., a combination of $\left(B_{Z}^{\text{RB}},B_{Z}^{\text{RG}},B_{Z}^{\text{BG}}\right)$. For example, in the first step, we could find the outcome $\left(A_{X}^{R},A_{X}^{B},A_{X}^{G}\right)=\left(0,1,0\right)$ and, based on this measurement, then apply $B_{Z}^{\text{RG}}$, which shares an even number of sites with the red and green plaquettes and a single site with the blue one, thus fixing the state into the desired target codespace. This procedure is inverted for switching from [[7,1,3]] to [[15,1,3]]: First, the Z-stabilizers connecting the 2D color code instance with the yellow cell are measured, i.e., $\left(B_{Z}^{\text{RB}},B_{Z}^{\text{RG}},B_{Z}^{\text{BG}}\right)$, and a combination of $\left(A_{X}^{R},A_{X}^{B},A_{X}^{G}\right)$ is applied. The respective quantum feedback obeys a lookup table–like logic as summarized in Materials and Methods.

### MF FT code switching

The main idea behind MF code switching is to map the desired stabilizer information to an auxiliary register, but instead of measuring and providing classical feedback, a quantum feedback operation is directly applied with controlled Pauli gates as part of the quantum algorithm itself. The entropy is then removed by resetting the auxiliary qubits or replacing them with fresh auxiliary qubits. In the following, we translate the above scheme for FT code switching to an MF setting, which poses several challenges: (i) The stabilizer information has to be coherently transferred to the auxiliary register in a reliable way, (ii) the randomly initialized stabilizer value has to be distinguished from a single error that flipped a given syndrome bit, and (iii) the coherent feedback operation has to be FT.

The first challenge can be resolved by using suitable logical auxiliary qubits. The set of target stabilizers can then be mapped to these logical auxiliary qubits with purely transversal operations and subsequently stored on a subset of physical qubits, as indicated in Fig. 2. The logical auxiliary qubit has to be chosen such that it shares specific stabilizers with the initial and the target code. It has to be a +1-eigenstate of the target stabilizers to ascertain the desired stabilizer values. Additionally, the logical auxiliary qubit has to share the respective complementary Pauli-type stabilizers of the data qubit to avoid unwanted back-propagation of Pauli operators onto the data qubits. Here, we use the logical $|\;0\rangle_{L}$ of the seven-qubit color code for switching from [[15, 1, 3]] to [[7, 1, 3]]. This code shares the three X-stabilizers with the target code as well as the Z-plaquettes with the initial tetrahedral code and is, therefore, a suitable candidate for MF code switching. Analogously, we use three [[8, 3, 2]] code instances in the $|\;+++\;\rangle_{L}$ state, as defined in Fig. 3C, for the inverse switching direction enabling the reliable copying of all desired stabilizer operators, which is discussed further in Materials and Methods. Both of these auxiliary codes have to be initialized in an MF manner themselves. We build on circuits for the logical [[7, 1, 3]] code, developed in (*21*, *22*), and construct circuits for the MF initialization of the [[8, 3, 2]] auxiliary qubits, given in Materials and Methods.

The second challenge is to identify if an error on a data qubit has propagated onto the auxiliary register and inverted the extracted stabilizer value. Without any additional information, it is not possible to identify these errors because the state is initialized randomly in a +1- or −1-eigenstate of the stabilizer. However, we can distinguish the randomly initialized stabilizer value from these potential errors by comparing pairs of opposing faces belonging to the same cell, which should agree in the fault-free case (*29*). The syndrome is coherently updated with Toffoli gates, which flips the respective syndrome bit if two opposing faces disagree.

**Fig. 4.**
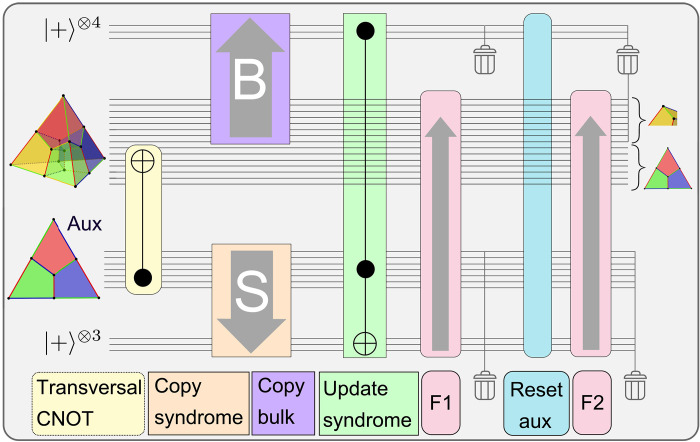
High-level circuit for MF fault-tolerant code switching. Sketch of the circuit scheme for an FT implementation of MF code switching protocols for switching from the [[15, 1, 3]] code to the seven-qubit color code [[7, 1, 3]]. After initializing a logical auxiliary qubit in $|0\rangle_{L}$ on the [[7, 1, 3]] code, the auxiliary qubit is coupled to the data qubits with a transversal CNOT gate (yellow), which effectively maps the stabilizers from the data to the auxiliary qubit. The corresponding syndrome information is transferred to a set of physical qubits afterward (orange). In parallel, we map the stabilizer values of the set of opposite faces (violet), which belong to the yellow cell of the tetrahedron, to an auxiliary register. These opposing faces should agree with their respective counterpart, so the syndrome is updated coherently with a Toffoli gate (green), i.e., it is flipped if there is a disagreement between opposing faces. The respective feedback operation is split into two parts, namely, feedback 1 (F1) and F2 (pink), and the full auxiliary register is reset in between (blue), which includes all of the previous steps (yellow, orange, violet, and green).

**Fig. 5.**
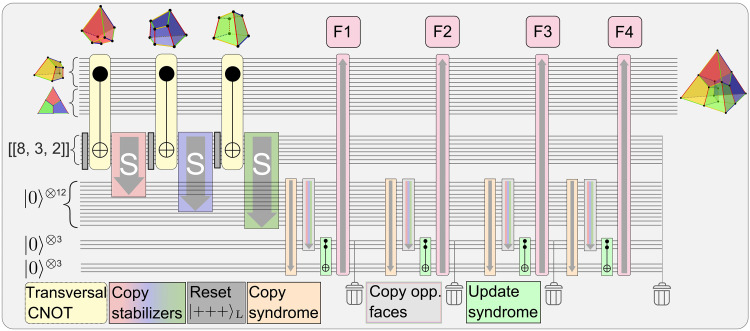
Schematic circuit for MF FT switching from the [[7, 1, 3]] to the [[15, 1, 3]] code. We use a similar strategy as for the inverse switching direction: First, we initialize a logical auxiliary qubit in the $|\;+++\;\rangle_{L}$ state of the [[8,3,2]] code (gray) using the circuit shown in fig. S1. Then, we couple those qubits belonging to the red cell to this encoded auxiliary register with a transversal CNOT gate (yellow) and copy pairs of opposing X-faces of this cell to a register of physical qubits, which are initially prepared in ∣0〉 (pink). We repeat this procedure for the blue and green cells (blue and green). Then, the syndrome information is transferred to a set of physical qubits (orange), as well as the opposing faces belonging to the same cell (gray). Last, the agreement of opposing faces within the same cell is checked by updating the extracted syndrome accordingly with a Toffoli gate (green). The quantum feedback operation (F1 to F4) is implemented in four steps with a reset of the updated syndrome in between (pink).

Last, the coherent quantum feedback operation has to be applied to a set of data qubits. State-of-the-art MF protocols for QEC (*20*–*22*, *24*, *30*) rely on multiqubit Toffoli gates to implement a lookup table feedback operation for small codes. But, in contrast to QEC, we can implement this feedback operation in an iterative manner, only relying on two-qubit controlled Pauli operations, which is discussed further in Materials and Methods. These switching operations correspond to multiple successive two-qubit gates with the same auxiliary control qubit but different data target qubits. The overall feedback operation is split into several parts to achieve fault tolerance. In between, we reset the syndrome and repeat the previous steps for coherent syndrome extraction. Otherwise, a single fault on a qubit storing one of the syndrome bits would propagate onto all four participating qubits and result in a logical failure. Figure 4 illustrates the MF FT switching procedure and summarizes the protocol for switching from [[15, 1, 3]] to [[7, 1, 3]]. The scheme for MF FT switching in the inverse direction is constructed conceptually analogously and is discussed further in Materials and Methods (Fig. 5).

### Numerical results

**Fig. 6.**
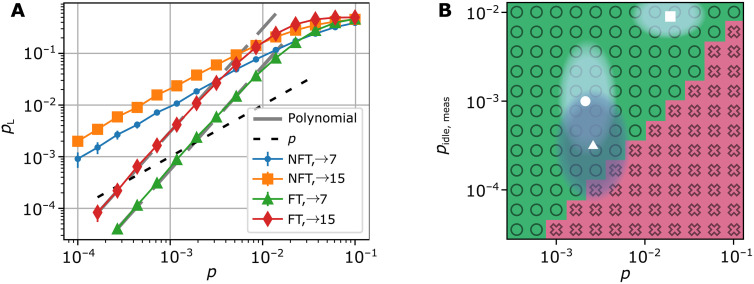
MF FT code switching and logical operations. (**A**) Logical failure rates for switching from the [[15, 1, 3]] to the [[7, 1, 3]] code and the inverse direction for the FT and non-FT MF protocol, averaged over different logical input states. All two-qubit gate components infer an error with the error rate *p* and any single-qubit gate operation introduces an error with a probability $\frac{p}{10}$. The gray dashed lines correspond to the approximated polynomial (Eq. 1) with the coefficients given in table S2. The black dashed line is the physical error rate, indicating a breakeven point for the FT schemes at $p\approx 3\times 10^{-4}$. (**B**) Difference in the logical error rate between the measurement-based and the MF protocol for the FT $H_{L}$ gate on the tetrahedral [[15, 1, 3]] code, operated above threshold in the regime of current experimental capabilities. The MF protocol achieves lower logical failure rates in the area depicted in green with circles, while the measurement-based version yields lower logical failure rates in the area shown in pink with crosses. The symbols indicate parameter regimes demonstrated in experiments with trapped ions in static traps (□) (*12*, *15*), shuttling-based traps (○) (*16*), and with neutral atoms in tweezer arrays (Δ) (*5*, *42*–*44*).

**Fig. 7.**
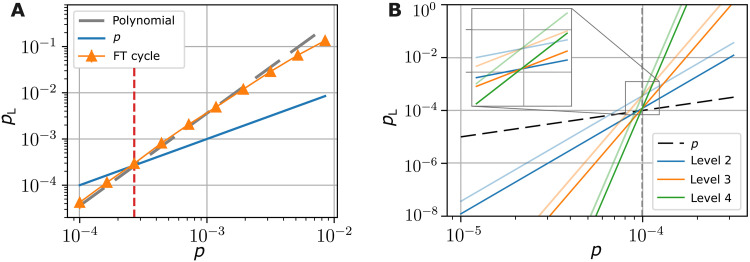
MF FT code switching cycle and approximated scaling of the logical T gate. (**A**) Logical error rate for a full switching cycle, starting and ending in the [[7, 1, 3]] code. The gray dashed line corresponds to the summed polynomials of each individual switching step. We find a breakeven point at approximately $p_{\text{th}}=2.6\times 10^{-4}$ as indicated by the red dashed line. (**B**) Approximated error polynomials of the logical error rates for the logical T gate (darker colored) and the logical Toffoli gate (lighter colored) for concatenation levels l=2,3,and4, based on the coefficients summarized in table S2 and the polynomials given in table S3 for small physical error rates *p*. The level *l* Toffoli gate error rates dominate the logical failure rates of the logical level (*l* − 1) T gate, and we find a pseudothreshold of $p_{\text{th}}\approx 1\times 10^{-4}$ for both logical operations (gray dashed vertical line).

We perform Monte Carlo simulations (*31*) and implement circuit level noise, as specified in Materials and Methods. Here, we focus first on a single-parameter noise model, where every two-qubit gate in the circuit introduces an error with probability *p* and each single-qubit operation is faulty with probability $\frac{p}{10}$. In this setting, we find that the FT schemes outperform their non-FT counterpart below physical error rates of $p\approx 2\times 10^{-2}$ and $p\approx 10^{-2}$ in the two switching directions, respectively, as shown in Fig. 6A. We estimate the performance of a logical gate, which does not have a transversal implementation on the given code by simulating a full cycle of switching back and forth and extract a breakeven point as shown in Fig. 7 at $p_{\text{th}}\approx 2.6\times 10^{-4}$. Furthermore, we compare the performance of the FT MF logical gate to an FT measurement-based version of this protocol (*27*) and determine which scheme achieves lower logical failure rates. To this end, we introduce an additional parameter $p_{\text{idle},\text{meas}}$, which indicates the error rate on idling data qubits during measurements. We identify a large parameter regime where the MF logical operation outperforms the measurement-based version, shown in green in Fig. 6B.

### Scalability of MF FT universal gates

Scaling up an FT quantum computing architecture to high-distance codes is a crucial step toward building practical, large-scale quantum computers that require low error rates (*32*). However, this task presents major challenges in the measurement-based setting, as qubit overhead, computational complexity, and hardware requirements increase substantially. Code concatenation offers a powerful method for constructing high-distance codes from smaller ones because the failure probability is suppressed doubly exponentially below the threshold with each layer of concatenation while maintaining polynomial time decoding complexity (*6*, *33*), at the cost of exponentially growing qubit overhead. In the following, we scale the presented MF FT protocols to high-distance codes by concatenating the [[7, 1, 3]] color code with itself and combining this with code switching, thus effectively giving access to the logical T gate for the concatenated [[7, 1, 3]] code.

As a first step, we estimate the leading order contributions to the logical failure rate for the initial, nonconcatenated code. The noisy two-qubit gates and the three-qubit Toffoli gates dominate the total logical failure rate because the number of two-qubit gates is orders of magnitude larger than that of single-qubit operations. This allows us to approximate the effective error polynomial, as discussed further in Materials and Methods, as

$$p_{L}\approx c_{2}p_{2}^{2}+c_{2,\text{toff}}p_{2}p_{\text{toff}}+c_{\text{toff}}p_{\text{toff}}^{2}+O\left(p^{3}\right)$$ (1)

The coefficients $c_{2},c_{2,\text{toff}}$, and $c_{\text{toff}}$ correspond to the number of weight 2 faults on the specified components (two-qubit gates, Toffoli gates) that lead to a logical failure, and $p_{2}\;\text{and}\;p_{\text{toff}}$ are the error rates on the respective circuit component. We determine the coefficients $c_{2}$ and $c_{2,\text{toff}}$ for the different protocols by deterministically placing all weight 2 fault configurations and counting the number of faults leading to a failure, as summarized in table S2. We find that the approximated polynomial in Eq. 1 fits the logical failure rates, as shown in Fig. 6A.

**Fig. 8.**
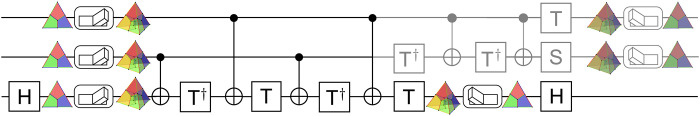
Logical operations on the concatenated [[7, 1, 3]] code. Decomposition of a Toffoli gate into single- and two-qubit gates. If the control qubits are reset after this gate, we do not need to execute the operations shown in gray. We additionally need to switch from the [[7, 1, 3]] to the tetrahedral [[15, 1, 3]] code before applying the non-Clifford T gate in the concatenated regime where each line corresponds to a logical [[7, 1, 3]] color code qubit. Note that all operations except the first and last H gates can be executed transversally on the [[15, 1, 3]] code, making the CCZ gate transversal for this code.

Now, we concatenate our scheme with the seven-qubit color code by replacing each physical qubit with another encoded [[7, 1, 3]]code. Each operation in our previous circuit then corresponds to a logical operation, as for example a physical controlled-NOT (CNOT) gate is translated into a transversal two-qubit gate ${\text{CNOT}}^{⊗7}$. Analogously, each physical error rate in Eq. 1 is replaced by the respective logical gate error rate, e.g., the physical two-qubit gate error rate $p_{2}$ is replaced by the transversal CNOT gate error rate $p_{2}^{\left(1\right)}$, where the superscript (1) indicates the level of concatenation. However, the Toffoli gate cannot be simply translated in the same way to the next concatenation level because it involves non-Clifford operations, which are not natively FT on the seven-qubit color code. The concatenated Toffoli gate can be realized by including code switching steps, as illustrated in Fig. 8.

Continuing to concatenate with the [[7, 1, 3]] code up to concatenation level l+1, we find that the noisiest components of the level l+1 logical T gate are the Toffoli gates. These Toffoli gates are themselves dominated by code switching steps on concatenation level *l* and, thus, the Toffoli gates of level *l* and so on. This, in turn, means, that the pseudothreshold of the logical operations of level l>1 is approximately given by the pseudothreshold of the concatenated Toffoli gate of level l≥1, as shown in Fig. 7. It is shifted to a slightly lower value as compared to that of the logical T_L_ of level 1 because the logical Toffoli gate now contains multiple code switching steps. However, we can estimate a lower bound of the pseudothreshold of the concatenated logical gate of level l>1 of $p_{\text{th}}^{l>1}\approx 1\times 10^{-4}$, based on the approximated effective error polynomials summarized in table S3.

In summary, we have constructed a toolbox for implementing any single logical operation fault-tolerantly and MF, which is scalable to larger code distances by concatenating the seven-qubit color code with itself and introducing the MF FT switch to realize logical Toffoli gates. However, running algorithms fault-tolerantly requires an additional building block, namely, QEC (*34*). Previous analyses on how to integrate QEC into a quantum algorithm (*33*, *35*, *36*) have been extended to concatenated codes (*37*, *38*), and recent works have shown that O(1) rounds of stabilizer extraction for each logical operations can be sufficient for specific FT quantum algorithms (*39*). Recent schemes for FT MF QEC (*22*) rely on Toffoli gates to implement corrections according to a lookup table based quantum feedback for the [[7, 1, 3]] code. We can integrate this scheme into our framework by concatenating the code with itself and including the MF FT switch for each logical Toffoli gate. Note that the MF QEC cycles coherently implement quantum feedback without the need of classical information processing. The cost of one QEC round below the pseudothreshold for concatenation level l>1 is much smaller than the cost of a logical T gate on the concatenated [[7, 1, 3]] code, as discussed in Materials and Methods. Therefore, the pseudothreshold of the combined block of this logical operation followed by QEC is still approximately given by the pseudothreshold of the bare logical operation.

## DISCUSSION

The presented MF and FT implementation of a universal gate set provides a route toward scalable FT quantum computing. Previous works on universal gate sets by means of concatenated codes (*37*, *40*, *41*) rely on the concatenation of different code types with complementary sets of gates. These require at least 49 and up to 105 physical qubits to realize a universal gate set for a distance-3 code, while, in our approach, 35 qubits are sufficient, provided qubit reset is available, which reduces the experimental requirements substantially. Remarkably, the pseudothresholds of our protocols are competitive and lie in between the lower and upper bounds indicated in these works (*37*).

Our schemes provide a feasible and scalable approach for MF FT universal quantum computing. They are built on heavily parallelizable physical operations, which can be implemented efficiently in experimental platforms that offer long-range connectivity between qubits. Neutral atom platforms, for example, have demonstrated massively parallelized Clifford operations (*42*) as well as shuttling of entire logical qubits (*5*), which are key building blocks of our protocols. Furthermore, mid-circuit measurements and real-time feedback are still experimentally demanding due to relatively slow measurements, while single- and two-qubit gate fidelities are high (*5*, *42*–*44*). These features make neutral atom platforms an ideal candidate for MF protocols and concatenated code constructions (*45*). Complementary to neutral atom platforms, also trapped-ion quantum processors have demonstrated the capabilities required for handling concatenated codes in 2D architectures (*46*, *47*), and shuttling-based approaches are, in principle, able to host the presented code constructions (*13*, *16*, *48*, *49*). Also, superconducting platforms with long-range couplers are advancing toward the realization of nonlocal connectivity (*50*), while spin-qubit quantum computing architectures have shown progress along these lines, leveraging shuttling-based techniques (*51*, *52*).

Tailoring of the theoretical proposal to a given experimental platform is expected to further increase the logical success rates. This includes the adaptation to a biased noise setting, which is present in various experimental architectures (*5*, *12*, *43*) and might simplify the presented protocols, substantially reducing the hardware requirements. The implementation of natively supported multiqubit gates (*42*) could further mitigate hardware limitations. Additionally, the determination of optimal thresholds for concatenated quantum codes, as well as the extent of possible improvements to circuits and schemes, remains an open problem. Here, in particular, examining to which extent the integration of repetition code–based elements in the coherent quantum feedback loop (*23*, *24*) or coherent readout of an overcomplete stabilizer set (*20*, *22*) will be able to improve the threshold is an interesting future research extension. This includes exploring the potential trade-off between qubit resources and performance, by investigating protocols that might require a higher number of auxiliary qubits than in the present work but possibly offer increased thresholds.

Optimizing the integration of QEC into a logical algorithm in this concatenated setting (*37*, *39*) by investigating how often and on which concatenation levels (MF) QEC should be carried out offers the potential for improved logical error rates. Overall, our findings outline a practical pathway toward fully scalable FT quantum computing, leveraging a completely MF approach that makes our method feasible for various experimental state-of-the-art quantum hardware platforms.

## MATERIALS AND METHODS

### FT code switching

It is possible to transfer encoded information between the [[7, 1, 3]] and [[15, 1, 3]] codes because they correspond to two gauges of the same subsystem code (*29*). A subsystem code is defined by its gauge group G, which describes a general subgroup of the *n*-qubit Pauli group (*27*, *29*, *53*). The gauge group G of the tetrahedral subsystem code is generated by all independent X- and Z-type faces of the tetrahedral structure shown in Fig. 3B. The subsystem’s stabilizer group S⊆G is the center of G, and it is generated by those elements commuting with all other elements in G, which are the weight 8 cells $B_{\sigma }^{R},B_{\sigma }^{B},B_{\sigma }^{G},B_{\sigma }^{Y}$ with σ= X and Z. Compared to the stabilizers of the tetrahedral [[15, 1, 3]] stabilizer code as defined in Fig. 3B, the Z-stabilizers of the subsystem code are not defined on the 10 independent faces of the code, but only on the four weight 8 cells. On the tetrahedral [[15, 1, 3]] stabilizer code, the gauge of the subsystem is, therefore, fixed such that the codestate is not only a +1-eigenstate of the weight 8 cells but also of the Z-faces within the tetrahedron. In the regime of the seven-qubit color code [[7, 1, 3]], in addition to the cells, also, the three weight 4 X- and Z-faces, as shown in Fig. 3A, are fulfilled. Figure 5 shows the high-level circuit scheme for MF FT switching from the 2D [[7, 1, 3]] code to the 3D [[15, 1, 3]] code. Analogously to the inverse direction discussed in Fig. 2, we have to use a suitable code for the logical auxiliary qubits. Here, we choose three [[8, 3, 2]] codes. The red, blue, and green cells of the tetrahedron are each mapped to one instance of the [[8, 3, 2]] code. This code shares the Z-plaquettes of the target [[15, 1, 3]] code, and it is also a +1-eigenstate of the weight 8 X-volume operators defined on the cells of the subsystem and the initial code and can, therefore, be used to extract the target stabilizers. Here, we require four quantum feedback operation steps instead of two, as we did for the inverse switching direction. For switching from [[15, 1, 3]] to [[7, 1, 3]], we only included the feedback acting on the [[7, 1, 3]] instance and could disregard the bulk because this is reinitialized afterward. However, for switching from [[7, 1, 3]] to [[15, 1, 3]], the full weight 4 quantum feedback has to be applied directly to fix the gauge correctly.

### Construction of feedback operation

For switching between two codes, we can construct the quantum feedback operation in an iterative manner, which is different to the approach for QEC. We first consider switching from the [[15, 1, 3]] to the [[7, 1, 3]] code. If a qubit storing a certain syndrome bit is in the ∣1〉 state, then a certain Pauli plaquette has to be applied: If the qubit storing the syndrome bit $A_{X}^{R}$ is in ∣1〉, then we apply the Z-face $B_{Z}^{\text{BG}}$; if $A_{X}^{B}$ is in ∣1〉, then we apply the Z-face $B_{Z}^{\text{RG}}$; and if $A_{X}^{G}$ is in ∣1〉, then we apply the Z-face $B_{Z}^{\text{RB}}$. If several syndrome bits are in the ∣1〉 state, then the combination of the Pauli plaquettes is applied, which effectively flips some data qubits twice, thus implementing an identity operation on a subset of physical qubits. Note that, in practice, we only apply the respective operations on those data qubits, which encode the target [[7, 1, 3]] code and leave the qubits forming the yellow cell untouched. Before switching back, we reinitialize the yellow cell using the circuit shown in fig. S1. For the inverse switching direction, we use the same table but interchange the right and left column, e.g., if the syndrome bit storing the syndrome bit $B_{Z}^{\text{BG}}$ is in ∣1〉, then we apply the X-face $A_{X}^{R}$.

### Numerical methods

We perform Monte Carlo simulations to determine the logical failure rates of our protocols (*31*). Every component in the circuit is implemented by, first, applying the ideal operation followed by an error *E* with a given probability *p*. Specifically, we implement a depolarizing channel after each single- and two-qubit gate. With probabilities $p_{1}$ and $p_{2}$ one of the errors in the sets $E_{1}$ and $E_{2}$ is applied and we can define the error channels as

$$\begin{array}{c} E_{1}\left(\rho \right)=\left(1-p_{1}\right)\rho +\frac{p_{1}}{3}\sum_{i=1}^{3}E_{1}^{i}\rho E_{1}^{i}\\ E_{2}\left(\rho \right)=\left(1-p_{2}\right)\rho +\frac{p_{2}}{15}\sum_{i=1}^{15}E_{2}^{i}\rho E_{2}^{i} \end{array}$$ (2)

with $E_{1}^{k}\in \left\{\;X,Y,Z\;\right\}$, for k=1,2,3 and $E_{2}^{k}\in \{\text{IX},\text{XI},\text{XX},\text{IY},\text{YI},\text{YY},\text{IZ},\text{ZI},\text{ZZ},\text{XY},\text{YX},\text{XZ},\text{ZX},\text{YZ},\text{ZY}\}$ for k=1,…,15. Furthermore, we initialize and measure all qubits in the Z-basis and simulate faults on these components by applying X-flips after and before the respective operation with a given probability $p_{\text{init}}$ and $p_{\text{meas}}$. Additionally, idling qubits may dephase during measurements, which we model with the error channel

$$\begin{array}{c} E_{\text{idle},\text{meas}}\left(\rho \right)=\left(1-p_{\text{idle},\text{meas}}\right)\rho +p_{\text{idle},\text{meas}}\mathrm{Z\rho Z} \end{array}$$ (3)

We simulate a simple single-parameter noise model, where $p≔p_{2}=10\cdot p_{\text{init}}=10\cdot p_{\text{meas}}=10\cdot p_{1}$ and $p_{\text{idle},\text{meas}}=0$ for the results shown in Fig. 6A. We include dephasing during measurements for the comparison to the measurement-based protocol as indicated on the *y* axis in Fig. 6B.

Furthermore, we decompose the Toffoli gates into single- and two-qubit gates, as shown Fig. 8. If at least one of the two control qubits is not used afterward, then we do not need to apply the last six components shown in gray. We simulate the decomposed Toffoli gate that we use in our protocol for all eight possible binary input states and determine the probability of the target qubit being flipped. We perform a linear fit for each input state and average the obtained slope. For error probabilities $p≔p_{2}=10p_{1}$ on single- and two-qubit gates, we find

$$p_{\text{toff}}=2.88\left(10\right)\cdot p$$ (4)

Analogously, we also determine the probability of flipping the respective target qubit for two consecutive Toffoli gates that share one control and the target qubit, as used in parts of our MF FT switching protocols. In this case, we find

$$p_{2\text{toffs}}=5.12\left(23\right)\cdot p<2\cdot p_{\text{toff}}$$ (5)

We therefore simulate errors on each Toffoli gate by flipping the target qubit with probability $p_{\text{toff}}$, which slightly overestimates the total Toffoli gate error rate. Note that the controlled-controlled-Z (CCZ) gate is a native gate on Rydberg platforms (*54*, *55*), which would allow for a more efficient implementation of the constructed protocols.

### Effective error polynomial and fault-path counting

We estimate the leading order contributions to the logical failure rate of a protocol, such as switching or state initialization, for the initial, nonconcatenated code. For small physical error rates, these are

$$\begin{array}{c} p_{L}=c_{2}p_{2}^{2}+c_{1}p_{1}^{2}+c_{\text{toff}}p_{\text{toff}}^{2}+c_{\text{init}}p_{\text{init}}^{2}+c_{1,2}p_{1}p_{2}+c_{2,\text{toff}}p_{2}p_{\text{toff}}\\ +c_{2,\text{init}}p_{2}p_{\text{init}}+c_{1,\text{toff}}p_{1}p_{\text{toff}}+c_{1,\text{init}}p_{1}p_{\text{init}}+c_{\text{init},\text{toff}}p_{\text{init}}p_{\text{toff}}+O\left(p^{3}\right) \end{array}$$ (6)

where the coefficients $c_{i,j}$ with i,j={1,2,toff,init} correspond to the number of weight 2 faults on the specified components (single- and two-qubit gates, Toffoli gates, and physical qubit initializations) that lead to a logical failure, and $p_{1},p_{2},p_{\text{toff}},\text{and}\;p_{\text{init}}$ are the error rates on the respective circuit components. However, the number of two-qubit gates in our MF FT code switching protocols is orders of magnitude larger than that of single-qubit gates and physical qubit initializations. Therefore, we estimate $c_{1},c_{\text{init}},c_{1,\text{init}},c_{1,\text{toff}},\;\text{and}\;c_{\text{init},\text{toff}}\ll c_{2}$ and, in the following, neglect these coefficients in the above error polynomial. Furthermore, we consider smaller error rates on single-qubit gates and initializations than on two-qubit gates (*15*) and approximate $p_{1}=p_{\text{init}}=\frac{p_{2}}{10}$ in our simulations. In this regime, we therefore also approximate that the contributions from error configurations with one fault on a two-qubit gate and another fault on a single-qubit gate or a physical qubit initialization scaling with $p_{1}$ and $p_{\text{init}}$ are negligible. With these approximations, we find in leading order

$$p_{L}\approx c_{2}p_{2}^{2}+c_{2,\text{toff}}p_{2}p_{\text{toff}}+c_{\text{toff}}p_{\text{toff}}^{2}+O\left(p^{3}\right)$$ (7)

For the switching and state initialization protocols, we determine the coefficients in the error polynomial by deterministically placing all possible weight 2 configurations on the specified type of component, summarized in table S2. We plot Eq. 7 with the determined coefficients given in table S2 for each switching direction and compare it to the logical failure rate obtained from Monte Carlo simulations, which is shown in Fig. 6A. We find that the logical failure rate and the determined polynomial agree within 6% below $p=10^{-3}$.

### Concatenated error polynomial

Next, we extend this approach to the building blocks required for FT quantum computing, namely, the initialization of a logical qubit in the [[7, 1, 3]] code, final projective measurements, the logical gates $H_{L}$, $T_{L}$, ${\text{CNOT}}_{L}$, and Toffoli gates on the seven-qubit color code, as well as switching in both directions and rounds of error correction, which have to be performed between logical operations in an algorithm (*33*–*37*, *39*). Table S3 summarizes the dominating leading order contributions to the logical failure rate for each block in the column labeled “level 1.” The logical failure rates for operations that have a natively transversal implementation on the [[7, 1, 3]] code, i.e., projective measurements and the H and CNOT gates, generalize in a straight-forward way to the next concatenation level: For seven physically executed gates, there are maximally $\left(\begin{array}{c} 7\\ 2 \end{array}\right)$ noncorrectable error configuration occurring with a probability $p^{\left(0\right)2}$, where $p^{\left(0\right)}$ is the physical gate error rate. Analogously for concatenation level l+1, the final logical error rate is given by $\left(\begin{array}{c} 7\\ 2 \end{array}\right)\cdot p^{\left(l\right)2}$ with the failure probability $p^{\left(l\right)}$ on the next lower concatenation level (*37*, *56*). Furthermore, we find that faults on the CNOT and Toffoli gates dominate the total rate $p_{L}$ for switching between codes on the first level of concatenation, while contributions from faulty single-qubit gate operations are negligible (Fig. 6A). We follow the same strategy to estimate the logical error rates of the remaining building blocks of qubit initialization and QEC: The total logical error rate is dominated by the two-qubit and Toffoli gate error rates, and we neglect the remaining parts of the polynomial. Note that the coefficient $c_{\text{toff}}^{\left(\text{init}\right)}=0$ for logical qubit initialization because it is only one Toffoli gate in the respective circuit.

The lowest weight *w* of an uncorrectable error configuration on a code that is constructed by concatenating two codes of distance $d_{1}$ and $d_{2}$ is given by

$$w=\frac{d^{\prime }+1}{2}=\frac{d_{1}+1}{2}\cdot \frac{d_{2}+1}{2}\Rightarrow d^{\prime }=\frac{1}{2}\cdot \left(d_{1}d_{2}+d_{1}+d_{2}-1\right)$$ (8)

where $d^{\prime }$ is the distance of the resulting concatenated code.

Comparing the different logical error rates of the first level of concatenation, we see that the logical error rate of any logical operation that includes a switching step is dominated by this switching procedure because this has a much higher error rate than the transversal CNOT or single-qubit gates. Specifically, we need to switch in both directions once to implement the logical T gate on the seven-qubit color code. The logical error rate of this operation $T_{L}^{\left(1\right)}$ is, therefore, approximately given by the error rates of the individual switching steps $p_{\leftarrow +\to }^{\left(1\right)}\approx p_{\leftarrow }^{\left(1\right)}+p_{\to }^{\left(1\right)}$. This approximation holds if one switching direction has much larger coefficients in the error polynomial than the other direction, which is the case here as summarized in table S2. Additionally, we verify this approximation by simulating a complete cycle of switching and compare this to the summed polynomials of each direction, as shown in Fig. 7A.

### Scaling of the logical failure rate for concatenated scheme

The Toffoli gate on the first level of concatenation also contains switching steps as illustrated in Fig. 4. We consider two versions of the Toffoli gate: the reduced Toffoli gate, where the control qubits are not used afterward and we only apply the operations shown in black, and the full Toffoli gate, which includes the full decomposition and we also execute the operations depicted in gray. If each qubit is an encoded [[7, 1, 3]] code before applying the Toffoli gate, then we have to switch to the [[15, 1, 3]] code after the first $H_{L}$ gate to apply the logical $T_{L}$ and the transversal ${\text{CNOT}}_{L}$ gates and, again, switch back to the [[7, 1, 3]] code in the end before the final $H_{L}$ gate. The logical error rate for the Toffoli gate is, therefore, also dominated by the switching procedure and can be approximated by summing up the individual switching error rates. Note that summing up all individual switching error rates on control and target qubits of the logical Toffoli gate overestimates the final probability of flipping only the respective target qubit.

We now determine the dominant contributions to the logical failure rate for concatenation level l+1 for the not inherently transversal logical gates of the [[7, 1, 3]] code. The rate of the full Toffoli gate on level 2 is again dominated by the switching procedure, i.e., the probabilities $p_{\to }^{\left(2\right)}$ and $p_{\leftarrow }^{\left(2\right)}$, which, in turn, are dominated by the logical Toffoli gate of level 1. Iterating this to level l+1, we find that the logical operations of concatenation level l+1 will always be dominated by the Toffoli gate error rate because this includes the most switching steps and, therefore, the noisiest parts of the respective logical operation. Also, compiling our circuits into three-qubit gates, which are natively supported on neutral atom (*54*, *55*) and some trapped-ion platforms (*57*), reduces the overhead in physical gate operations by only $O\left(7^{l}\right)$ gate operations. However, the overall logical error rate will not change significantly, as it is still dominated by the switching steps that have to be performed. Figure 7B shows the resulting polynomial with the leading order contributions to the logical failure rate, as given in the third column of table S3, for the logical concatenated $T_{L}$ and Toffoli gates. We find a similar pseudothreshold at $p_{\text{th}}\approx 1\times 10^{-4}$ for both protocols because the noisy Toffoli gates dominate the effective total logical error rate.

### Cost of QEC rounds

Like the MF FT code switching protocols, also, the QEC blocks of concatenation level l+1 are dominated by the logical Toffoli gate error rate, as this presents the component with the largest error rate. Considering protocol (*22*), we find an upper bound of the dominant coefficient $c_{\text{toff}}^{\left(\text{QEC}\right)}$ of the logical failure rate for one round of QEC to be

$$c_{\text{toff}}^{\left(\text{QEC}\right)}\leq \left(\begin{array}{c} \#\text{Toffoli}\;\text{gates}\\ 2 \end{array}\right)=\left(\begin{array}{c} 21\\ 2 \end{array}\right)\ll c_{\text{toff}}^{\left(\leftarrow \right)}+c_{\text{toff}}^{\left(\to \right)}$$ (9)

The cost of one round of QEC below the pseudothreshold for concatenation level l>1 is, therefore, much smaller than the cost of a logical T gate on the concatenated [[7, 1, 3]] code.

Note that the scheme presented in (*21*) may leave an X-error and a Z-error on different data qubits after one round of QEC. This error configuration is in general not correctable after applying the T gate because X-errors are mapped to a superposition of Pauli errors.

### Resource analysis and circuits

**Fig. 9.**
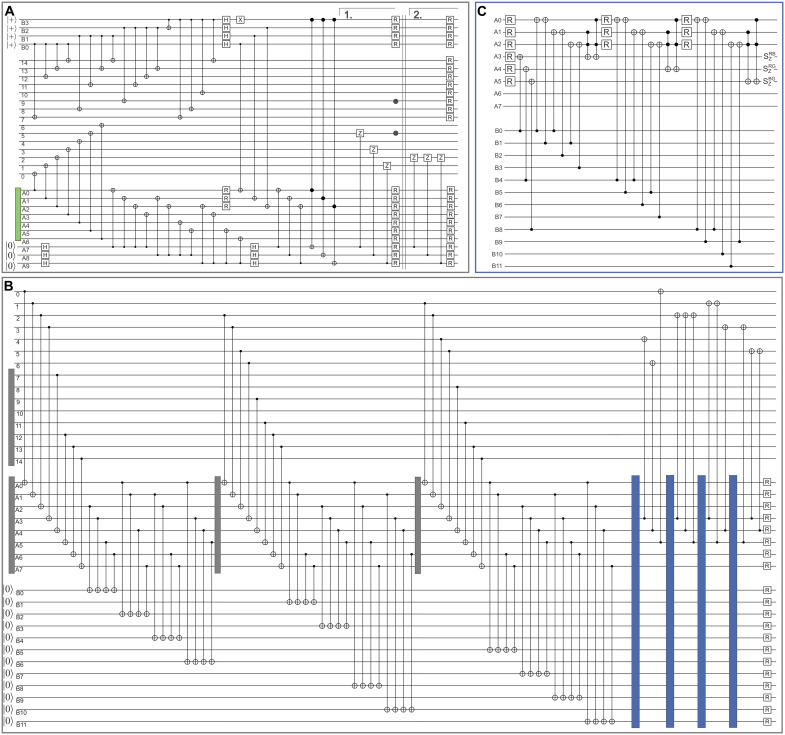
Circuits for MF FT switching between the [[15, 1, 3]] and the [[7, 1, 3]] code. (**A**) Switching from [[15, 1, 3]] to [[7, 1, 3]]. First, all shown gates up to the double line are executed. Then, we start from the beginning and, again, perform all gates up to bracket number 1 and jump into the second bracket 2 to execute the last three CZ gates. The operation R refers to qubit reset, which is either done by reinitializing the physical qubit in the ∣0〉 state or replacing it with a fresh qubit. The green box corresponds to the MF encoding of the logical $|0\rangle_{L}$ state on the [[7,1,3]] color code (*21*) as shown in fig. S1. (**B**) Switching from [[7, 1, 3]] to [[15, 1, 3]]. The gray boxes correspond to the MF initialization of the logical auxiliary state $|\;+++\;\rangle_{L}$ shown in fig. S1. The blue boxes correspond to the circuit shown in (C). (**C**) Circuit for updating the syndrome based on agreement check. Qubits A0, A1, and A2 are used to check the agreement of opposing faces by copying both respective syndrome bits onto the same qubit. Qubits A4, A5, and A6 store the syndrome information, which is updated with two Toffoli gates each.

**Fig. 10.**
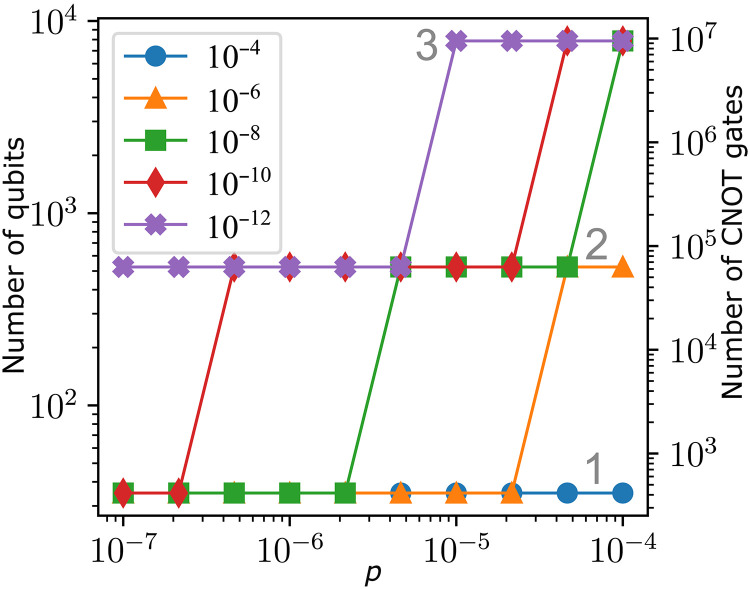
Resource overhead for the T_L_ gate. We determine the required level of concatenation to reach certain target logical error rates, indicated in the figure legend, for the logical $T_{L}$ gate for a given physical error rate *p*. We then recursively calculate the numbers of two-qubit gates and qubits for the required concatenation level. The gray numbers 1, 2, and 3 indicate the concatenation levels 1, 2, and 3, respectively.

Table S4 summarizes the required resources for the constructed MF FT protocols in terms of qubit count, number of two- and three-qubit gates. Figure 9 shows the explicit circuits that we implement for switching between the tetrahedral [[15, 1, 3]] code and the [[7, 1, 3]] code. Here, the reset-operation R includes the reinitialization of the respective physical qubit or the substitution with a fresh auxiliary qubit in a pure state. Based on the numbers of required two-qubit gates and qubits, we can estimate the resources that are needed at a given level of concatenation. For each new layer, every physical qubit is replaced by seven physical qubits, and every physical CNOT gate is replaced by seven CNOT gates. For each Toffoli gate, we need to perform at most three switching operations that each contain the number of gates and qubits required for the next lower level. The number of two-qubit gates $C_{l}$ and the number of physical qubits $Q_{l}$ at concatenation level *l* are, therefore, approximately given by

$$\begin{array}{c} C_{l}\approx 7\cdot C_{l-1}+3t\cdot C_{l-1}\\ Q_{l}\approx 7\cdot Q_{l-1}+t_{q}\cdot Q_{l-1} \end{array}$$ (10)

where t=48 is the number of Toffoli gates and $t_{q}=8$ is the number of distinct qubits that participate in parallel in Toffoli gates in the considered circuit. Here, the intuition is the following: One full code switching cycle on a level 1 logical qubit requires $Q_{1}=35$ physical qubits. To go to the next higher level, we replace each of the 35 physical qubits with a level 1 logical qubit consisting of seven physical qubits. During the code switching process, at most eight qubits are involved in Toffoli gates at the same time. Thus, we have to perform code switching on eight level 1 qubits, resulting in additional 8×35 physical qubits. Note that, in principle, one could use less additional physical qubits for the switching on the level 1 qubits, but we neglect this for simplicity. Replacing again all physical qubits by level 1 logical qubits and taking into account additional qubits for switching then lead to Eq. 10. Note that this formula is overestimating the number of qubits that is needed at a certain level of concatenation because we assumed that qubits are used in a sequential manner. This means that some qubits are idling while switching steps are performed on other sets of qubits. The exact number of required qubits depends on the scheduling and parallelization capabilities of the experimental platform. Given the polynomial in table S3, we can infer the level of concatenation that is needed for a certain physical error rate *p* to reach a certain logical error rate $p_{L}$. With this, we now estimate the overhead in gates and qubits that is required to reach a certain logical error rate for the logical $T_{L}$ gate, which is shown in Fig. 10.
