# Supplementary material for: Measurement-free, scalable, and fault-tolerant universal quantum computing
Source: Sci Adv. 2025 Aug 13;11(33):eadv2590. doi: 10.1126/sciadv.adv2590 (PMC13155506; doi:10.1126/sciadv.adv2590)
Supplement: Supplementary file 1 — Supplementary Text Fig. S1 Tables S1 to S4 [file sciadv.adv2590_sm.pdf]

Science Advances
AAAS

Supplementary Materials for

Measurement-free, scalable, and fault-tolerant universal quantum computing

Friederike Butt *et al.*

Corresponding author: Friederike Butt, friederike.butt@rwth-aachen.de

Sci. Adv. **11**, eadv2590 (2025)
DOI: 10.1126/sciadv.adv2590

This PDF file includes:

Supplementary Text
Fig. S1
Tables S1 to S4

Supplementary Results

MF FT initialization of logical auxiliary qubits

To ensure fault tolerance for switching from $[[7,1,3]]$ to $[[15,1,3]]$ we have to verify that no weight-2 X-error and no weight-2 Z-error is present on the encoded logical auxiliary state. In this switching direction, we make use of an auxiliary $[[8,3,2]]$, as specified in figure 6C. We construct a MF FT circuit for the initialization of the logical $[[8,3,2]]$ qubit in the $|{+}{+}{+}\rangle_L$-state by extending the non-FT encoding (59) and adding a verification. First, we prepare two weight-4 GHZ-states on separate sets of qubits and entangle these for the non-FT encoding. A weight-two Z-error is equivalent to a logical Z_L on the auxiliary qubit and would propagate onto two data qubits when the auxiliary and data register are coupled with the transversal CNOT-gate, as illustrated in figure 7. We can correct this weight-2 Z-error by mapping two complementary logical X_L operators to the auxiliary system by building on a flag-qubit based scheme (*22*), as shown in Supplementary figure S1 in the green box. The two logical operators are chosen such that only the dangerous weight-2 Z-errors anticommute with both of them. In the end we apply a Toffoli-type feedback operation so that a correction is only applied if both extracted operators are in the $|1\rangle$-state. Furthermore, a weight-2 X-error on the auxiliary register would propagate onto the second auxiliary register storing the syndrome information bits, similarly to X-errors on data qubits. We find that there are only two inequivalent weight-2 error configurations that can result from a single fault in the non-FT encoding by placing a fault at every possible location in the circuit. We can align one of these error configurations such that it flips the extracted syndrome bits either exactly twice or not at all. The second dangerous error configuration is corrected by extracting the instantaneous stabilizers to an auxiliary system and applying a Toffoli-type feedback operation (blue box in Supplementary figure S1) before entangling the two separate GHZ-states.

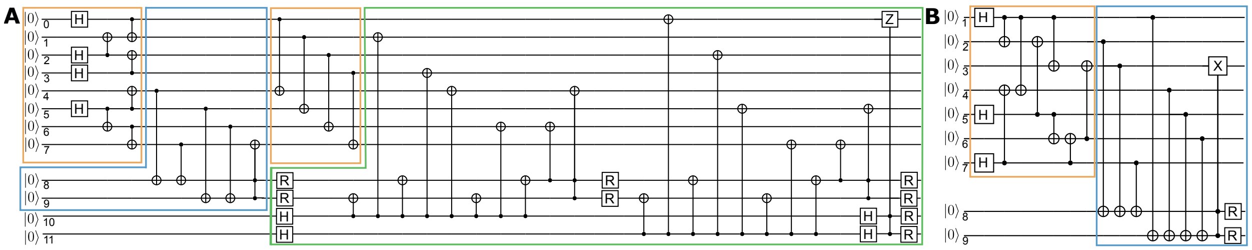

Figure S1: **Full circuit for MF FT initialization of logical auxiliary qubits. A** The $|{+}{+}{+}\rangle_L$-state of the $[[8,3,2]]$ code can be initialized by, first, preparing two GHZ-states (left orange box) and entangling these (right orange box). We perform a verification before entangling the two GHZ-states (blue) in order to detect potentially dangerous weight-2 X-errors. Finally, we need to verify that no weight-2 Z-error, which could result from a single fault in the circuit, is present on the qubit register. To this end, we extract two complementary logical X-operators which are chosen such that they anticommute with all potentially dangerous Z-error configurations (green box). **B** The $|0\rangle_L$ on the $[[7,1,3]]$ code can be initialized MF in a similar way by, first, implementing a non-FT encoding (orange), and then mapping suitable operators onto two physical auxiliary qubits and applying a Toffoli-gate (blue) (*21, 22*).

syndrome (A_X^R, A_X^G, A_X^B)	switching operation
(1, 0, 0)	$Z_2Z_5Z_{11}Z_{13}$
(0, 1, 0)	$Z_2Z_3Z_{12}Z_{13}$
(0, 0, 1)	$Z_1Z_2Z_{13}Z_{14}$
(1, 1, 0)	$Z_3Z_5Z_{11}Z_{12}$
(1, 0, 1)	$Z_1Z_5Z_{11}Z_{14}$
(0, 1, 1)	$Z_1Z_3Z_{12}Z_{14}$
(1, 1, 1)	$Z_1Z_2Z_3Z_5Z_{11}Z_{12}Z_{13}Z_{14}$
syndrome $(B_Z^{BG}, B_Z^{RB}, B_Z^{RG})$	switching operation
(1, 0, 0)	$X_0X_1X_2X_3$
(0, 1, 0)	$X_1X_2X_4X_5$
(0, 0, 1)	$X_2X_3X_5X_6$
(1, 1, 0)	$X_0X_3X_4X_5$
(1, 0, 1)	$X_0X_1X_5X_6$
(0, 1, 1)	$X_1X_3X_4X_6$
(1, 1, 1)	$X_0X_2X_4X_6$

Table S1: **Feedback operation for switching between [[15, 1, 3]] and [[7, 1, 3]].** For each possible switching syndrome indicated in the left column, we apply the respective Pauli-operation on the right.

Protocol	coefficient	# weight-2 faults $\lvert+\rangle_L/\lvert 0\rangle_L$
$[[15,1,3]] \rightarrow [[7,1,3]]$	$c_2^{(\rightarrow)}$	300.05/386.60
	$c_{2,\text{toff}}^{(\rightarrow)}$	69.06/18.94
	$c_{\text{toff}}^{(\rightarrow)}$	9/0
$[[15,1,3]] \leftarrow [[7,1,3]]$	$c_2^{(\leftarrow)}$	1832.53/490.38
	$c_{2,\text{toff}}^{(\leftarrow)}$	178.67/469.33
	$c_{\text{toff}}^{(\leftarrow)}$	3/1220
$\lvert 0\rangle_L^{[[7,1,3]]}$	$c_2^{(\text{init})}$	27.88
	$c_{2,\text{toff}}^{(\text{init})}$	8.27

Table S2: **Coefficients from fault-path counting for different protocols.** We determine the number of weight-2 error configurations that lead to a logical failure for two input states $\lvert+\rangle_L$ (left) and $\lvert 0\rangle_L$ (right) for each block, indicated by the two numbers in the right column. The index "2" means that two faults are placed on different two-qubit gates, the index "2, toff" means that one fault is placed on a two-qubit gate and one fault on a Toffoli-gate and "toff" indicates that two faults are placed on different Toffoli-gates. We divide the obtained number of logical failures by 15 if an error is placed on a two-qubit gate, after deterministically placing all 15 Pauli fault-combinations after two-qubit gates and counting each configuration individually. Note that we do not yet take the effective Toffoli-gate error rate, given in equation (4) into account.

Operation	physical	level 1	level $l+1$
Initialization $\|0\rangle$	$\frac{p_2^{(0)}}{10}$	$c_2^{(\text{init})} p_2^{(0)2} + c_{2,\text{toff}}^{(\text{init})} p_2^{(0)} p_{\text{toff}}^{(0)} + c_{\text{init,toff}}^{(\text{init})} \frac{p_2^{(0)}}{10} p_{\text{toff}}^{(0)}$	$\approx c_{\text{init,toff}}^{(\text{init})} p_{\text{init}}^{(l)} p_{\text{toff}}^{(l)}$
Measurement	$\frac{p_2^{(0)}}{10}$	$\binom{7}{2}(\frac{p_2^{(0)}}{10})^2$	$\binom{7}{2}(\frac{p_2^{(l)}}{10})^2 \ll p_{\text{toff}}^{(l)2}$
H-gate	$\frac{p_2^{(0)}}{10}$	$\binom{7}{2}(\frac{p_2^{(0)}}{10})^2$	$\binom{7}{2}(\frac{p_2^{(l)}}{10})^2 \ll p_{\text{toff}}^{(l)2}$
T-gate	$\frac{p_2^{(0)}}{10}$	$\approx p_{\leftarrow}^{(1)} + p_{\rightarrow}^{(1)}$	$\approx (c_{\text{toff}}^{(\leftarrow)} + c_{\text{toff}}^{(\rightarrow)}) p_{\text{toff}}^{(l)2}$
CNOT-gate	$p_2^{(0)}$	$\binom{7}{2} p_2^{(0)2}$	$\binom{7}{2} p_2^{(l)2} \ll p_{\text{toff}}^{(l)2}$
Red. Toffoli-gate	$p_{\text{toff,red}}^{(0)}$	$\approx 3p_{\leftarrow}^{(1)} + p_{\rightarrow}^{(1)}$	$\approx (3c_{\text{toff}}^{(\leftarrow)} + c_{\text{toff}}^{(\rightarrow)}) p_{\text{toff}}^{(l)2}$
Toffoli-gate	$p_{\text{toff}}^{(0)}$	$\approx 3p_{\leftarrow}^{(1)} + 3p_{\rightarrow}^{(1)}$	$\approx 3(c_{\text{toff}}^{(\leftarrow)} + c_{\text{toff}}^{(\rightarrow)}) p_{\text{toff}}^{(l)2}$
$15 \rightarrow 7$	-	$p_{\rightarrow}^{(1)} = c_2^{(\rightarrow)} p_2^{(0)2} + c_{2,\text{toff}}^{(\rightarrow)} p_2^{(0)} p_{\text{toff}}^{(0)} + c_{\text{toff}}^{(\rightarrow)} p_{\text{toff}}^{(0)2}$	$\approx c_{\text{toff}}^{(\rightarrow)} p_{\text{toff}}^{(l)2}$
$15 \leftarrow 7$	-	$p_{\leftarrow}^{(1)} = c_2^{(\leftarrow)} p_2^{(0)2} + c_{2,\text{toff}}^{(\leftarrow)} p_2^{(0)} p_{\text{toff}}^{(0)} + c_{\text{toff}}^{(\leftarrow)} p_{\text{toff}}^{(0)2}$	$\approx c_{\text{toff}}^{(\leftarrow)} p_{\text{toff}}^{(l)2}$
QEC round	-	$p_{\text{QEC}}^{(1)} = c_2^{(\text{QEC})} p_2^{(0)2} + c_{2,\text{toff}}^{(\text{QEC})} p_2^{(0)} p_{\text{toff}}^{(0)} + c_{\text{toff}}^{(\text{QEC})} p_{\text{toff}}^{(0)2}$	$\approx c_{\text{toff}}^{(\text{QEC})} p_{\text{toff}}^{(l)2}$

Table S3: **Approximated error rates for operations on the concatenated code.** For each operation indicated on the left, we estimate the leading order contributions to the logical failure rates for concatenation level l. The error rates for the non-concatenated case (second column) correspond to the physical error rates on each component. Each physical qubit is then replaced by a $[[7,1,3]]$ logical qubit in concatenation level 1 (third column). Here, the indicated superscript of the coefficient c specifies the considered building block and the subscript specifies the considered component, e.g. $c_2^{(\text{init})}$ is the number of faults only on two-qubit gates during the (logical) qubit initialization. The level of concatenation is indicated by the superscript on the error rates, as for example $p_2^{(0)}$ is the error rate of the physical two-qubit gates. We determine the specified coefficients with fault-path counting, which are explicitly given in Supplementary table S2. We include the approximation discussed in the main text Sec. and neglect parts of the full polynomial. The most costly component in our circuits is the concatenated Toffoli-gate, because it includes several switching operations, each with a large gate overhead. We therefore find that for high levels of concatenation, the logical error rates of the non-transversal logical operations is dominated by the effective Toffoli-gate error rate $p_{\text{toff}}^{(l)} \approx (3[c_{\text{toff}}^{\rightarrow} + c_{\text{toff}}^{\leftarrow}])^{2^l-1} \cdot p_{\text{toff}}^{(1)2^l}$.

Protocol	CNOTs	Toffolis	qubits
$[[15,1,3]] \rightarrow [[7,1,3]]$	120	8	29/53
$[[15,1,3]] \leftarrow [[7,1,3]]$	296	40	35/131
$\|0\rangle_L^{[[7,1,3]]}$	15	1	9
$\|+++\rangle_L^{[[8,3,2]]}$	32	4	12/16
$T_{[[7,1,3]]}$	416	48	35/169

Table S4: **Resources required for MF FT building blocks.** Number of CNOT- and Toffoli-gates and the number of physical qubits required for the protocols indicated on the left. This includes MF switching, the initialization of the logical auxiliary qubits and the logical T-gate on the 2D color code. The two given numbers for the qubit count correspond to the protocol with qubit resets (left) and without qubit resets (right).
